# Viruses and Skin Cancer

**DOI:** 10.3390/ijms22105399

**Published:** 2021-05-20

**Authors:** Sara Becerril, Roberto Corchado-Cobos, Natalia García-Sancha, Leonor Revelles, David Revilla, Tatiana Ugalde, Concepción Román-Curto, Jesús Pérez-Losada, Javier Cañueto

**Affiliations:** 1 Department of Dermatology, Complejo Asistencial Universitario de Salamanca, Paseo San Vicente 58-182, 37007 Salamanca, Spain; sarabecerrilandres@gmail.com (S.B.); leonor_lrp@hotmail.com (L.R.); drevillan@saludcastillayleon.es (D.R.); cromancurto@gmail.com (C.R.-C.); 2 Laboratory 7, Instituto de Biología Molecular y Celular del Cáncer (IBMCC)-Centro de Investigación del Cáncer (CIC)-CSIC, Campus Miguel de Unamuno s/n, 37007 Salamanca, Spain; rober.corchado@usal.es (R.C.-C.); nataliagarciasancha@usal.es (N.G.-S.); ugaldecatarinella.tatiana@usal.es (T.U.); jperezlosada@usal.es (J.P.-L.); 3 Instituto de Investigación Biomédica de Salamanca (IBSAL), Complejo Asistencial Universitario de Salamanca, Paseo San Vicente 58-182, Hospital Virgen de la Vega, 10ª Planta, 37007 Salamanca, Spain

**Keywords:** HPV, cervical cancer, cutaneous squamous cell carcinoma, Merkel cell carcinoma, Kaposi’s sarcoma

## Abstract

Advances in virology and skin cancer over recent decades have produced achievements that have been recognized not only in the field of dermatology, but also in other areas of medicine. They have modified the therapeutic and preventive solutions that can be offered to some patients and represent a significant step forward in our knowledge of the biology of skin cancer. In this paper, we review the viral agents responsible for different types of skin cancer, especially for solid skin tumors. We focus on human papillomavirus and squamous cell cancers, Merkel cell polyomavirus and Merkel cell carcinoma, and human herpesvirus 8 and Kaposi’s sarcoma.

## 1. Introduction

In 1910, Peyton Rous demonstrated that a spontaneous spindle cell sarcoma derived from a Plymouth Rock hen could be transmitted to healthy chickens using filtered cell-free tumor extracts [1]. Ellermann and Bang characterized a transmissible filtrate that gave rise to leukemia in chickens three years earlier, but the avian sarcoma induced by Rous sarcoma virus (RSV), as the agent responsible for this disease was called, was a genuine cancer, similar to the malignant solid tumors seen in mammals [2]. At first, the scientific community did not universally accept the idea that tumors could be produced by transmissible agents. However, this momentous discovery became the basis for a new field of research on tumors induced by viral agents and led to Peyton Rous being awarded the Nobel Prize over half a century later. Throughout the twentieth century, a plethora of significant advances in this field established it as one of the most productive areas in oncology.

The International Agency for Research on Cancer (IARC) divides carcinogenic agents, including biological agents, into five categories according to their potential carcinogenicity [3]. Group 1 includes agents for which there is sufficient evidence of carcinogenicity both in humans and experimental animals (such as human papillomavirus types 16, 18, 31, 33, 35, 39, 45, 51, 52, 56, 58, 59, and HHV8). Group 2A includes probably carcinogenic agents for which limited evidence in humans and sufficient evidence in experimental animals exists (as is the case for Merkel cell polyomavirus and HPV68). Group 2B includes possibly carcinogenetic agents (such as HPV5 and HPV8). In this review, we discuss the viruses that are most closely related to the development of different types of skin cancer by summing up their pathogenesis, clinical features, and prevention strategies.

## 2. Human Papillomavirus

HPVs are small non-enveloped viruses with an icosahedral capsid in the Papillomaviridae family (PV). More than 200 serotypes have been identified so far [3]. HPVs have been classified into five major genera (α, β, γ, ν, and µ), where α-HPV and β-HPV are those more clearly implicated in human diseases: α-HPVs are closely associated with mucosal and skin infections and they have been linked to cervical carcinoma and benign viral warts, while β-HPVs are associated with skin infections and they have been linked to cutaneous squamous cell carcinomas (see Table 1). Furthermore, α-HPVs can be classified as low-risk, such as HPV-6 and HPV-11, or high-risk, such as HPV-16 and HPV-18. 

Classification of HPVs is based on the nucleotide sequence of the ORF coding for capsid protein L1. HPV types belonging to different genera have less than 60% similarity within the L1 part of the genome. Different viral species within a genus share between 60 and 70% similarity. A novel HPV type has less than 90% similarity to any other HPV type [3].

HPV infection targets undifferentiated keratinocytes in the basal layer of the epithelia, at the cutaneous and mucosal levels [3]. Although all HPVs replicate inside the epithelia, they are subdivided based on their capacity to infect cutaneous or mucosal keratinocytes.

### 2.1. Historical Perspective

In the mid-1970s, some authors began to postulate that HPVs could be involved in the development of cervical cancer [4,5,6]. In 1976, Meisels and Fortin suggested that the appearance of koilocytes in the cervical smear of some patients might indicate presence of an HPV infection [7]. This idea was supported by the identification of HPV particles in dysplastic cervical lesions [8,9]. The first types of HPV to be isolated directly from cervical cancer biopsies, HPV16 [10] and HPV18 [11], were cloned in the beginning of the 1980s. 

More specifically, in 1983, the DNA of a type of a previously undescribed papillomavirus was identified in 60% of all cervical cancer biopsies [10]. The authors called it HPV16 and observed that it was not present in viral warts, leading them to conclude its specificity to cervical cancer [10]. In 1984, the same team found a new type of HPV in samples of genital cancer with cell lines derived from cervical cancer, which they called HPV18 [11]. They concluded that HPV16 and HPV18 were very closely associated with malignant tumors in the anogenital region since up to that point they had only detected HPV18 in malignant tumors and HPV16—in malignant tumors and high-risk lesions [11]. The significance of these findings led to Harald zur Hausen eventually being awarded the Nobel Prize in Physiology or Medicine in 2008. These two types of HPV are consistently found in over 70% of all cervical cancer biopsies globally [12].

By the end of the 1970s, some types of HPV were known to be involved in the development of verrucous lesions seen in epidermodysplasia verruciformis [13,14] and in the development of genital warts [15] and laryngeal papillomatosis (HPV6 and HPV11) [16]. In the beginning of the 1980s, DNA from HPV6 was found in penile and vulvar verrucous carcinomas (Buschke–Löwenstein tumor) [17], and HPV16 proved to be involved in the development of bowenoid papulosis [18], and it has also been associated with Bowen’s disease.

### 2.2. Structure and Pathogenicity Mechanisms

#### 2.2.1. Structure and Genome

**Figure 1:**
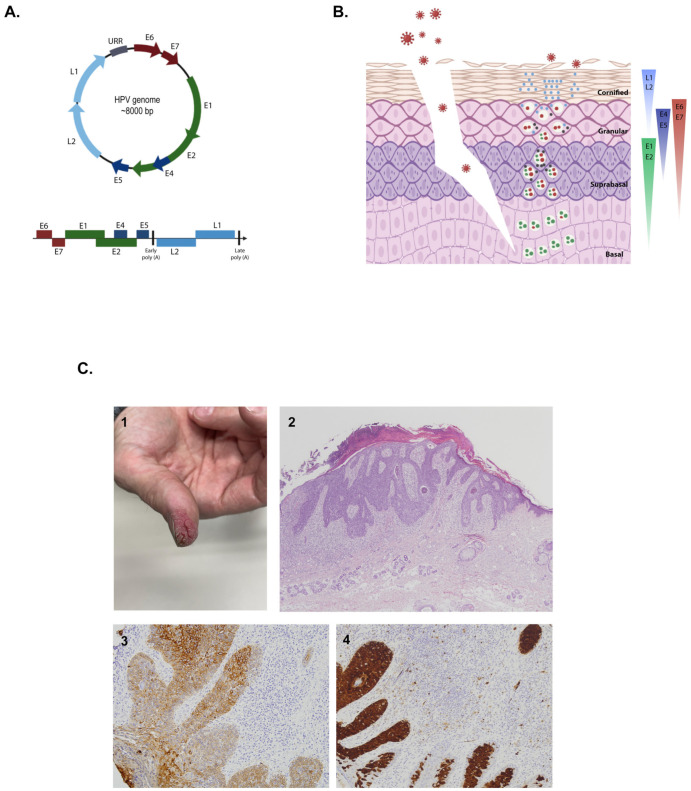
Human papillomavirus and cutaneous squamous cell dysplasia. (**A**) HPV genome and structure. (**B**) HPV infective cycle, which is associated with the differentiation process of the keratinocytes. (**C**) Bowen’s disease, which is a type of cutaneous squamous cell dysplasia: 1 shows the clinical aspect of a lesion, which consists of an erythematous plaque located on the first finger of one patient; 2 displays the histopathological aspect of Bowen’s disease; hyperkeratosis is evident, acanthosis with prominent dysplasia difficult to observe with this 40× magnification; 3 shows pan-cytokeratin staining; and 4 displays the p16 staining, which is associated with HPV infection. Bowen’s disease and cutaneous squamous cell carcinoma located on the fingers are related to HPV infection. All the schemes and photographs were generated and provided by authors. To generate (**A**,**B**), BioRender was used.

The genome of HPVs is made up of circular double-stranded DNA, approximately 8 kb in size (Figure 1A). The viral genomes of HPVs carry an average of eight major open-reading frames (ORFs) that are expressed from mRNA molecules transcribed from a single DNA strand [19]. Despite their small size, their molecular biology is notably complex.

The HPV genome is organized in three main regions: (1) an upstream regulatory region (URR), which contains transcriptional factor binding sites and regulates the gene expression of the virus; this region is not transcribed and acts as a promoter [20]; (2) an early transcription region (E), which contains six genes with multiple functions, including virus replication and cell transformation; the E1 and E2 genes in these regions are transcribed during the earliest stages while the E6 and E7 genes are more involved in the oncogenicity of the virus [3]; and (3) a late transcription region (L), which encodes proteins L1 and L2 that auto-assemble to form the capsid. In summary, three oncogenes (E5, E6, and E7) modulate the transformation process, two regulatory proteins (E1 and E2) modulate transcription and replication, and two structural proteins (L1 and L2) form the viral capsid [21,22]. The URR and proteins E1, E2, L1, and L2 are particularly well-conserved among the different members of the family [20,21,22].

#### 2.2.2. Infective Cycle

The infective cycle of the virus is linked to the differentiation program of the keratinocytes [3] (Figure 1B). The virion enters basal keratinocytes through microwounds or hair follicles [23,24]. Infection does not necessarily require the virus to be in the form of a virion as skin abrasion and exposure to the naked viral genome are able to recapitulate the complete natural history of the infection in different animal models [3]. The naked viral genome is incorporated into the nucleus of the cell after one complete mitotic cycle and replicates there as episomes. Genome integrity and correct segregation into daughter cells is ensured by the E1 and E2 viral proteins [25]. 

Under normal circumstances, as keratinocytes migrate upwards and begin the differentiation process, they stop replicating and undergo a series of changes until they form the corneal layer. The E6 and E7 proteins hijack the checkpoint mechanisms and allow the keratinocytes to enter a phase of uncontrolled proliferation [26,27]. More specifically, the E7 protein binds to retinoblastoma protein (pRb) and promotes its phosphorylation, causing the release of the E2F factor, which in turn induces the unscheduled reentry into the S-phase cell cycle [26,27,28], whereas the E6 protein induces degradation of p53. HPV E6 and E7 proteins are also involved in epigenetic modulation, chromatin remodeling, and miRNA expression, promoting deregulation of tumor suppressors and oncogenes and, consequently, keratinocyte transformation. Finally, the E5 protein promotes hyperproliferation, prevents apoptosis of infected cells, and is likely to facilitate malignant progression [29]. All the other proteins act as cofactors in this process. After keratinocyte differentiation, the E6 and E7 proteins are replaced by E1, E2, E4, and E5, and the viral copy number increases to thousands per cell [30]. Proteins E6 and E7 act as oncoproteins in high-risk, but not low-risk, types [19].

In the upper layers of the epithelium, viral gene expression shifts towards the L2 and L1 capsid proteins, which are targeted to the nucleus and auto-assemble into virions, encapsidating the viral genome [31]. Finally, viral release proceeds without cell lysis. In the URR, proteins E1, E2, L1, and L2 are the best-preserved elements among the various types of HPV. The gene L1 ORF is taken as a reference for the taxonomic classification of HPVs.

A reciprocal correlation between pRb and p16 has been established, which explains p16 overexpression in carcinomas and premalignant lesions of cervical cancer associated with HPV infection. In the case of β-HPVs, the activity related to proteins E6 and E7 is not the same as that in α-HPVs, but instead involves the degradation of Bcl-2. The hair follicle is regarded as the natural reservoir for cutaneous β-HPVs. HPV is present in hair follicles from different body sites including the scalp, eyebrows, arms, trunk, legs, and pubic region [32]. The β-HPV type spectrum in eyebrow hair follicles is to a significant degree representative of different body sites, suggesting a generalized infection of the entire skin. Eyebrow hairs have therefore been used as an easily collected marker in many recent epidemiological studies [33].

### 2.3. Clinical Symptoms, Prognosis, and Treatment

#### 2.3.1. Infection of the Anogenital Region

It is estimated that more than half of all sexually active people will be infected with HPV at some point in their lives. In most cases, HPV infection clears up spontaneously, without any associated health disorders. However, some lesions may develop into genital warts, precancerous lesions, and cancer. The HPV types that cause genital warts are different from those that cause anogenital cancer [34]. HPVs are associated with benign lesions (viral warts, including genital warts) and malignant lesions (anogenital cancer (particularly cervical cancer), oropharyngeal carcinomas, and cutaneous squamous cell carcinoma (cSCC), including penile and vulvar cSCC).

##### Benign Lesions: Genital Warts

Genital warts are the most common clinical symptom associated with HPV infection. In general terms, the diagnosis of genital warts is reached by visual inspection [34]. The HPV test is not recommended for the diagnosis of genital warts because the results are not confirmatory and do not guide the management of the patient [34]. Treatment is directed towards dealing with macroscopic lesions. Subclinical genital HPV infection typically clears spontaneously, so specific antiviral therapy is not recommended for its eradication [34]. Precancerous lesions are detected by cervical cancer screening (see below). 

There are multiple treatment options for genital warts (cryotherapy, imiquimod cream, podophyllotoxin, sinecatechins ointment) whose analysis lies beyond the scope of this review [34]. Women with genital warts do not need cytological (Pap) tests more frequently than do other women [34]. Although genital warts can be treated, such treatment does not eliminate the virus itself [34]. For this reason, it is common for genital warts to recur after treatment, especially within the first three months. Sexual activity should be avoided with new partners until the warts have gone [34]. HPV may remain present and can still be transmitted to partners even after the warts have disappeared. HPV can infect areas that are not covered by condoms, which therefore may not provide full protection against HPV [34]. Existing vaccines (for men and women) can prevent most cases of genital warts in persons who have not yet been exposed to HPV [34].

##### Malignant Lesions

###### Cervical Cancer

Epidemiology: Cervical cancer has an estimated incidence of 12,000 cases in the US, with 4000 estimated deaths in 2016 and an incidence of 7.7 per 100,000 people. It is, however, a huge health concern in developing counties. Cervical cancer is the fourth most common cancer affecting women worldwide after breast, colorectal, and lung cancers, with 604,127 new cases in 2020. It is also the fourth most common cause of cancer death (341,831 deaths in 2020) in women worldwide. Almost 70% of the global burden occurs in developing countries where it is the second most commonly diagnosed cancer and third leading cause of cancer death among women [35]. 

Oncogenic (high-risk) HPVs are responsible for almost all cases of cervical and anal cancer, and for a smaller fraction of cases of penile, vaginal, vulvar, and oropharyngeal cancers [12]. Progression from a precursor lesion generally takes more than 10 years without treatment, which makes it possible to instigate early treatment with screening programs [36]. 

Diagnosis: Clinical presentation depends mainly on the location and extent of disease. Precancerous changes or very early-stage disease are usually asymptomatic. Advanced stages can cause vaginal bleeding, foul smelling, vaginal discharge, dyspareunia, pelvic or back pain, and inguinal lymphadenopathy [37]. 

Colposcopy is a diagnostic procedure that can visually detect dysplastic changes and guide biopsies to obtain a histologic diagnosis [38]. The most common site of persistent HPV infection and cervical cancer development is the transformation zone between the columnar epithelium of the endocervix and squamous epithelium of the ectocervix [39,40]. 

After initial colposcopy, lesions are graded as cervical intraepithelial neoplasia (CIN) 1, 2, or 3 based on the degree of cellular and epithelial abnormality. The majority of CIN 1 are spontaneously cleared by the immune system; hence, surveillance rather than treatment is recommended. CIN 3 lesions represent a full-thickness neoplastic lesion with a high likelihood of progressing to cancer. CIN 2 lesions are intermediate, with up to 40% regressing if left untreated. The cornerstone of cervical cancer prevention is the detection of CIN 2 and CIN 3 lesions through screening and their eradication through treatment [38,41]. 

Treatment: In the era before colposcopy, the traditional treatments for CIN used to be conization and hysterectomy. After the introduction of colposcopy in the 1970s, more conservative treatments such as cryotherapy, electrocautery, and laser ablation were adopted. In the 1990s, an office-based excisional procedure known as loop electrosurgical excision procedure (LEEP) became available and was widely adopted [41,42,43]. Unnecessary cervical excisions must be avoided in young women because these procedures increase the risk of preterm birth and mid-trimester pregnancy loss [40,44]. 

Potential treatment modalities for invasive cancer include surgery (i.e., cone biopsy, trachelectomy, or hysterectomy); lymph node evaluation/dissection; radiotherapy (brachytherapy or systemic therapy) [40].

Prevention: The CDC recommends that cervical cancer screening should begin at the age of 25 years due to the low incidence of cervical cancer and limited utility of screening in younger women. HPV infection is very common in adolescents, in whom it typically clears up spontaneously [34]. Conventional or liquid-based cytological Pap tests are recommended, and screening can include DNA tests for high-risk HPV (HR-HPV) [34]. The cytopathological report is required to follow the Bethesda 2001 or LAST terminology, which defines three classes: (1) atypical squamous cells (ASCs); (2) low-grade squamous intraepithelial lesions (LSILs); (3) high-grade intraepithelial lesions (HSILs). Cervical cancer screening is generally based on cytopathological analyses and colposcopy [12].

Tests for different types of HPV are used for cervical cancer screening in conjunction with a Pap test, for the triage of abnormal cervical cytology results, and for follow-up after treatment of cervical precancers. These tests are only approved for use with cervical (i.e., not oral or anal) specimens. Testing for non-oncogenic HPV types (6 and 11) is not recommended. Tests for oncogenic types of HPV are now being incorporated into cervical cancer screening recommendations with Pap tests (co-testing) to reduce the need for follow-up visits by women over 30 years of age. Sexual partners do not need to be tested for HPV. In the future, oncogenic (high-risk) HPV tests might be considered for primary cervical cancer screening, but no such recommendation has so far been made by any medical organization [34]. 

A quadrivalent vaccine, which combines the L1 protein from HPV6/11/16/18, and a 9-valent vaccine, which contains the L1 protein of HPV6/11/16/18/31/33/45/52/58, are currently available; the bivalent vaccine is no longer marketed. All three vaccines protect against HPV16 and HPV18, which account for over 70% of cervical cancer cases. The quadrivalent and 9-valent vaccine also protect against other types of HPV, including those responsible for the development of genital warts. The success of cervical cancer screening programs is evident from the sharp decrease in cervical cancer incidence rates observed in the US between the 1970s and 2012 [36]. 

##### Penile and Vulvar Carcinoma

The majority of anal carcinomas, roughly 80% of cases, are caused by HPV. Men that have sex with men, HIV+ individuals and women with cervical or vulvar cancer are at greatest risk [40]. Clinical features of anal carcinoma may be variable, from bleeding to tenesmus, fecal incontinence, pain, etc. Screening for anal cancer should be offered to persons at high risk for developing anal cancer [40]. 

Vulvar SCCs tend to appear in postmenopausal women, and it begins as vulvar intraepithelial neoplasms [45,46,47]. The proportion of vulvar cSCCs attributed to HPV is variable depending on the studies [45]. Vulvar cSCCs tend to be nodular, sometimes ulcerated lesions, which may be exophytic, located in the vulvar area [45]. Beyond HPV, lichen sclerosus and other chronic inflammatory conditions are responsible for vulvar cSCC [47].

Penile SCC has been linked both to long-lasting inflammatory conditions (such as sclerotic and atrophic lichen) and to HPV infections [48]. HPV is found in more than half of the cases. Poor hygiene and lack of circumcision are also risk factors for penile SCC [48]. Despite the fact it is uncommon, it is a health concern in poor countries, where early diagnosis is rarely performed. Early diagnosis is important since it may avoid mutilating surgery. Penile SCC derives from intraepithelial neoplasia (PIN), similar to VIN and CIN [49]. It is important to rule out PIN in an erythematous plaque that persists in the glans, glans sulcus, and prepuce (erythroplasia of Queyrat). PIN may appear as millimetric smooth lesions either in the penile shaft or in the vulvar area, similar to plane warts, and is sometimes difficult to identify, so-called bowenoid papulosis [40].

Bowen’s disease consists of an intraepithelial dysplasia which may appear anywhere in the skin and that has been linked to HPV as well, e.g., to HPV56 [50] and HPV58 [51]. Those cases found in the skin of the genital area and around the nails are more likely to be associated with HPV [52,53,54,55].

##### Verrucous cSCC of the Anogenital Region: Buschke–Löwenstein Tumor

HPV has been observed in other forms of anogenital cancer, including verrucous cSCC of the anogenital region, which is also known as Buschke-Löwenstein tumor. From a histopathological perspective, this tumor presents an abundant proportion of clear cells, an exo–endophytic pattern, with tumor lobules that may show dyskeratotic cells, but generally display a low level of differentiation. It has been associated with HPV6 and HPV11 infection (which is responsible for genital warts) [36]. The reasons why this form of cSCC develops in patients at low risk of HPV infection are not completely understood and it is a rare variant of cSCC.

#### 2.3.2. Epidermodysplasia Verruciformis (Lewandowsky–Lutz Dysplasia)

In 1902, Lewandowsky and Lutz described a hereditary disease in which patients developed multiple verruca-like lesions and had an increased risk of developing cSCC [37]. This condition, which was called epidermodysplasia verruciformis (EV), or Lewandowsky–Lutz dysplasia, involves, from a clinical perspective, the appearance of lesions that can be classified into two types according to their phenotype: some patients develop lesions similar to flat warts; others develop lesions similar to tinea versicolor. Notably, 40% of all patients develop some type of cSCC (either in situ or invasive) towards the fourth decade of life, especially on areas exposed to ultraviolet radiation [37]. In the 1970s, viral particles were detected for the first time on the lesions of epidermodysplasia verruciformis, and the viruses were found to be similar to those of common warts [38,39].

At the end of the 1970s, DNA from two types of papillomavirus was identified in EV lesions [13,14]. Shortly after, both serotypes—HPV5 and HPV8—were found to be present in the cSCC of patients with EV [40,56]. The researchers concluded that at least some of the HPV-induced lesions in patients with EV could evolve to cSCC and, in fact, 90% of all cSCC cases in EV patients were found to be associated with these two HPV serotypes [40,56]. HPV5 and HPV8 are actually low-risk types for healthy individuals. In patients with EV, alterations have been found in two gene loci, EV1 and EV2, on chromosomes 17 and 2, respectively [57,58]. A mutation in one of the two genes in the EV1 region (TCM6 and TCM8), which encode the relevant proteins for the viral function, was found in 75% of patients with EV [59]. 

Patients with EV (1) have difficulties clearing their β-HPV infection; (2) do not have a higher risk of infection caused by bacteria and other viruses (including α-HPVs); (3) do not have a higher risk of developing other types of cancer; (4) feature UV radiation as a cofactor in the development of cSCC [59]. In short, when EVER1 and EVER2 operate properly, they interact with ZnT-1, which causes underregulation of AP-1 and prevents replication of the virus [60]. If one of those proteins does not work properly, overregulation of AP-1 promotes viral replication [60]. Regardless of the underlying mechanisms in the elevated susceptibility to β-HPV infection in these patients, the elevated susceptibility is significant in itself, as is the elevated risk that some of those HPV-induced lesions may evolve into cSCC.

#### 2.3.3. Cutaneous Squamous Cell Carcinoma 

Beta HPV types are etiological agents that are potentially involved in the development of non-melanoma skin cancer (NMSC) in immunocompromised patients (e.g., organ transplant recipients, OTRs) [61,62]. The NMSC rate in solid organ transplant recipients is much higher than in the general population, particularly in the case of patients with cSCC, who also have a greater risk of developing viral warts.

The higher incidence of keratinocyte carcinomas in immunocompromised patients points to a possible viral origin. A comparison with the rates in the general population shows that the risk of developing NMSC (particularly cSCC) is much higher in immunocompromised than in normal patients (65 times more in solid OTRs) [63]. Two thirds of all solid organ transplant recipients develop skin cancer within 24 months. The cumulative incidence of cSCC increases progressively and two thirds of the patients develop a second cSCC within the subsequent five years. By 15 years after their transplant, 90% of patients will have developed viral warts, and almost half of them (40%) will have developed some type of NMSC (particularly cSCC) [64,65,66]. However, the risk is not the same for all the types of transplants, being successively greater in liver, through kidney and lung, to heart. Cases of cSCC in solid OTRs are more aggressive [66,67], with a higher five-year recurrence and metastasis rate (39% vs. 15%) [67] and a mortality rate of 5% (vs. <1%) [66]. Ultimately, the higher incidence of keratinocyte carcinomas in immunocompromised patients suggests a viral origin [68]. 

Cases of cSCC in transplanted patients may exhibit clinical and morphological characteristics similar to those of viral warts (induced by HPV). Generally, cSCC coexists with viral warts, which suggests that persistent viral warts may evolve in these patients [69]. More importantly, the prevalence of β-HPV DNA in actinic keratosis and cSCC of transplanted patients is notably higher than in the general population [70,71]. Although the involvement of β-HPV in the etiology of cSCC in patients with EV has been established (as it probably also has been in immunocompromised patients), the contribution of β-HPV to the development of NMSC in the general population is still a matter of debate [72,73]. Although the genomes of some types of β-HPV are frequently detected in some cases of NMSC in healthy individuals, they can also be found in premalignant lesions [74] and even in healthy skin. However, epidemiological studies have shown that patients with a history of cSCC have a greater probability of being positive for β-HPV infection than do normal individuals [75]. 

The prevalence of β-HPV in actinic keratosis is higher than in cSCC, which suggests that β-HPV may play a role in the initial stages of carcinogenesis (contrary to what occurs with α-HPV). In addition, recent sequencing studies have failed to find HPV in cSCC, which contrasts with observations in cervical cancer. A transgenic mouse model provides evidence of the causal role of HPV in the development of skin cancer. Mice that express the region of HPV8 under the simultaneous control of human keratin-14 promoter develop skin cancer spontaneously [76]. STAT3 seems to play a role in this process [77], and UV radiation accelerates it [78]. Nevertheless, the contribution of β-HPV to the development of cSCC in the general population is controversial, although a meta-analysis seems to have established that β-HPV infection is a risk factor for the development of SCC in healthy individuals. A subgroup analysis has highlighted this association, particularly with HPV5 and HPV8 (which are involved in cSCC in patients with EV), but also with HPV17, HPV20, and HPV38 [79].

## 3. Merkel Cell Polyomavirus and Merkel Cell Carcinoma

### 3.1. Historical Perspective

In 1895, Friedrich Sigmund Merkel described skin cells that he called Tastzellen (“touch cells”). These cells were found in the basal layer of the skin, connected to nerve endings and present in hair follicles, on some mucosal surfaces, and in touch-sensitive areas, which explains why their function was initially thought to be due to touch sensation. In 1972, Toker described a tumor made up of small polygon-shaped and highly basophilic cells that were organized as trabeculae, which prompted the entity to be named trabecular carcinoma [80]. Five years later, Tang and Toker suggested that, due to the presence of neurosecretory granules in their ultrastructure, Merkel cells were the cellular origin of trabecular carcinoma [81]. Until 1990, Merkel cell carcinoma (MCC) was rarely diagnosed, but after the development of the CK20 antibody in the early 1990s, the diagnosis became increasingly common. When observed in H&E sections, MCC is composed of polygonal basophilic cells arranged in a trabecular layout. The cells are positive for CK20, synaptophysin, neuron-specific enolase, and T antigen [82]. 

After the development of cytokeratin 20 (CK20) as an antibody in the early 1990s, the diagnosis of MCC became simpler and more reliable, and the reported incidence of MCC increased significantly, partly explaining why this rate has tripled in the last 20 years [83]. Until the 2000s, there were almost no reliable studies about the impact of this form of NMSC. Advanced age, influence of UV radiation, and immunocompromised status are the three most important risk factors for developing MCC. These risk factors highlight it as a form of cancer requiring further research in the proportion of the elderly population that has been subject to the influence of UV radiation, as is the case in our community.

### 3.2. Structure and Pathogenicity Mechanisms

**Figure 2:**
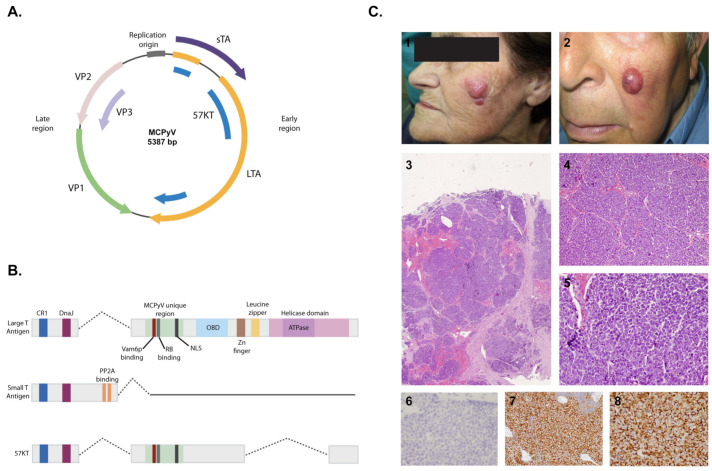
Merkel cell polyomavirus (MCPyV) and Merkel cell carcinoma (MCC). (**A**) MCPyV genome. (**B**) Proteins expressed by MCPyV. (**C**) Pictures 1 and 2 show examples of rapidly growing erythematous nodular lesions on the sun-exposed skin of elderly patients, consistent with Merkel cell carcinomas; 3–5 display the H&E aspect of MCC, in which polygonal blue cells are arranged in lobules with scattered mitosis; 6–8 show negative TTF1, as well as positive CK20 and chromogranin. All the schemes and photographs were generated and provided by authors. To generate (**A**,**B**), BioRender was used.

Merkel cell polyomavirus (MCPyV) is part of the polyomavirus family. As such, it has double-stranded DNA and a short circular genome. That of MCPyV is 5389 base pairs long and comprises early- and late-coding regions separated by a non-encoding regulatory region containing the origin of viral replication. The early genes, namely, the products of the T antigen locus (large and small T antigens), are expressed immediately upon infection and are generally involved in replication of the viral DNA genome (Figure 2A). Following DNA replication, the late region is transcriptionally activated to express gene products that are structural components of the viral capsid and contribute to progeny virion production during the late phase of infection.

The MCPyV genome is similar to other human polyomaviruses in that it includes a conserved replication origin, as well as opposing early and late gene regions. The early region contains small and large T antigens (sT and LT) and a 57 kT antigen (57kT). The late region expresses the major capsid protein viral protein 1 (VP1) and the minor capsid protein 2 and 3 (VP2 and VP3) antigens (Figure 2B). MCPyV is subject to pathogenicity mechanisms that are similar to other polyomaviruses and primarily utilize carbohydrates with sialic acid as primary receptors. It requires sulfated glycosaminoglycans (particularly heparan sulfate) for cellular attachment and association with sialic acid.

Polyomavirus origins of replication typically contain pentanucleotide consensus sequences that help direct assembly of LT proteins on the DNA by specifically binding the origin-binding domain (OBD), which initiates replication. Early region gene products of polyomavirus generally target cellular proteins that function in cell cycle regulation and tumor suppression. These gene products are designated as tumor antigens, or T antigens, so the early region is frequently referred to as the T antigen locus. The polyomavirus T antigens also perform key functions during the initiation of viral DNA synthesis. The MCPyV early region expresses three T antigen (LT, sT, and 57kT). All three share a short amino-terminus, but alternative splicing downstream of the first exon is ultimately responsible for determining their unique nature. The late region of the MCPyV genome contains open reading frames for VP1, VP2, and VP3. MCPyV LT is the main viral oncoproteins, while sT plays an accessory role in transformation.

### 3.3. Merkel Cell Carcinoma

#### 3.3.1. Epidemiology

MCC is a rare and often fatal neuroendocrine skin cancer. It has an incidence of approximately 1500 cases per year in the US, similar to those of cutaneous T cell lymphoma and dermatofibrosarcoma protuberans [83]. Although it accounts for fewer than 1% of all cases of skin cancer [84], it is the third most common cause of skin cancer death after melanoma and cSCC [85]. Its incidence has increased progressively in the US, going from 0.15 to 1.44 cases per 100,000 people over the three decades from 1981 to 2011 [85,86]. 

The population at risk includes the elderly and patients who are immunocompromised [87,88,89], either due to solid organ transplantation, HIV infection, or hematological disease [90,91]. This cancer is more common in elderly patients, with a peak incidence between the seventh and eighth decades of life [92]. The average age at diagnosis is 75 years, and only 5% of patients are younger than 50 years of age [93]. The relevance of polyomavirus [94] and UV radiation [95,96] has been pointed out in the tumorigenesis of MCC. 

#### 3.3.2. Clinical Features

The most common clinical features of MCC can be summarized in an acronym: AEIOU [82]: asymptomatic/lack of tenderness, expanding rapidly (≤three months), immunosuppression, older than age 50 years, and location on a UV-exposed site. Most (89%) primary MCCs exhibit three or more of these characteristics. There has been a significant increase in the incidence of MCC among immunocompromised patients, including patients with chronic lymphocytic leukemia [97] and solid organ transplantation recipients [82,98]. MCC is a disease that mainly affects elderly patients, with an average age of onset in men and women of 74 and 76 years, respectively [84]. It appears almost exclusively in Caucasian patients, and at sites that are exposed to UV radiation (Figure 2C). In fact, the prevalence of the disease is correlated with the proximity to the equator [82,84]. Interestingly, Merkel cells are more concentrated in sun-exposed than in non-exposed areas of the skin [99], and specific UV-induced mutations have been observed in the p53 gene of MCC [100]. As a whole, immunosuppression and UV radiation seem to be involved in the pathogenesis of MCC. The prognosis of MCC was initially considered to be poor, with a lower five-year survival rate than that of any other form of skin cancer, but the mechanisms underpinning its development were not well-known until approximately 10 years ago.

#### 3.3.3. Prognosis

This cancer has a higher five-year relative mortality rate than malignant melanoma (46% vs. 15%), and the cases presenting without clinical evidence of nodal disease have a 32% chance of microscopic nodal involvement [83]. In general terms, up to 37% of patients start with lymph node metastasis, and 6–12% present distant metastasis [82,84,92,96,101,102]. The management of MCC has been challenging due to the lack of prospective clinical studies and the scarcity of centers with experience or particular interest in this disease [103]. Consequently, the real impact of the incidence and mortality attributable to this type of skin cancer is not widely known.

#### 3.3.4. Treatment

Surgery is the treatment of choice for localized Merkel cell carcinoma. The recommended resection margins for MCC have not been assessed, although for functional reasons, 1–2 cm margins are recommended. 

Several retrospective studies have shown that adjuvant radiotherapy is very likely to have benefits for survival and is therefore recommended [104,105,106]. In MCC, adjuvant radiotherapy has proved useful for the local control of the disease, even when there are positive margins after resection of the primary tumor. In fact, it does not seem that the characteristics of the surgical margin influence the rate of disease recurrence after radiation therapy [107]. Occult lymph node disease is common in MCC, occurring in more than 25% of cases [108]. 

SLNB is recommended when possible regardless of the size of the primary tumor [109,110]. If micrometastases are observed, elective and therapeutic lymph node dissection is recommended, although no studies have reported a positive effect on long-term survival rates [109]. 

Avelumab is an anti-PD-L1 monoclonal antibody that showed a response rate of 32% in a phase 2 study of patients with metastatic MCC who had been refractory to conventional chemotherapy; 82% of those patients were still responding at a 10-month follow-up [111]. The data from this clinical assay accelerated the FDA’s approval of avelumab for the treatment of metastatic MCC. The FDA currently regards avelumab as the first-line treatment for disseminated MCC [112,113].

## 4. Human Herpesvirus 8 and Kaposi’s Sarcoma

### 4.1. Historical Perspective

In 1872, Moritz Kaposi described five patients who had what he called primary idiopathic pigmented sarcoma [114]. The patients were elderly and, according to his description, presented a tumor composed of disorganized endothelial cell proliferations forming blood-filled vascular clefts, but also containing areas of organized micro-neovascularization, often with an inflammatory infiltrate. Kaposi’s sarcoma (KS) was rescued from oblivion in the early 1980s. According to Kaposi’s description, the tumor particularly affects the elderly Mediterranean population (leading to it being referred to as “classic Kaposi”). Another variety of KS was subsequently found to be disproportionately common in areas of sub-Saharan Africa (before the HIV pandemic) and was assigned the name “endemic KS” [115]. In 1981, an unusually high incidence of KS cases was observed in homosexual men in the United States. These tumors were particularly aggressive, and the patients presented abnormally high rates of some opportunistic infections (such as a type of pneumocystis, initially called *Pneumocystis carinii*, but subsequently renamed as *Pneumocystis jirovecii*) [116,117,118,119]. In the 1990s, it was established that KS was caused by HH8 infection in patients with and without HIV [120]. 

### 4.2. Structure and Pathogenicity Mechanisms

#### 4.2.1. Structure

Kaposi’s sarcoma-associated herpesvirus (KSHV) is the eighth member of the herpesvirus family (HHV8) to be identified. Its genome is complex but similar to those of other human herpesviruses [121]. As with all members of the Herpesviridae group, it is a double-stranded DNA virus [122,123,124]. It is large and encapsidated, approximately 165 kb in length, and encodes approximately 100 genes. Like other herpesviruses, it has the ability to establish latency in the host. KSHV encodes at least 17 microRNAs and 87 open reading frames (ORFs), 14 of which code for cellular orthologues [121]. The KSHV virion is covered by a lipid bilayer and encodes five conserved herpesvirus glycoproteins plus a unique HHV8 glycoprotein associated with the lytic cycle of K8.1 and the gene products of ORFs 68 and 28 [125,126]. It also has a protein tegument and an icosahedral capsid.

#### 4.2.2. Infective Cycle

KSHV has a biphasic life cycle comprising latent and lytic infections [127]. Its DNA is found in more than 90% of spindle cells in late stages of KS, mostly in the latent phase [128]. Latency is characterized by a state of quiescence in which the viral genome persists as a circular episome attached to the host chromatin, with limited expression of viral transcripts and downregulated surface markers. On the other hand, the lytic cycle involves the expression of most genes and the production of viral particles [129]. Latency-associated nuclear antigen (LANA) is a protein with 1162 amino acids encoded by ORF73 which is responsible for the persistence of the virus.

HHV8 has broad cellular tropism and interacts with several cell proteins, including molecules such as heparan sulfate, integrins, DC-SIGN, and the heteromeric amino acid transporter (xCT) [130,131,132,133]. Entry into the host cell is a complex process in which the viral envelope glycoproteins bind to the cell surface receptors, then penetrate into the cytosol via direct fusion or internalization by endocytosis; this is followed by transport of the viral capsid to the nuclear periphery through intracellular signaling pathways and finally viral release into the nucleus [134].

Viral latency is associated with proteins such as LANA, vCyclin, vFLIP, vIRF4, kaposin, and viral microRNAs [135]. LANA (which may be detected immunohistochemically) is considered an essential protein for the persistence of KSHV episomes in infected cells during the latency phase because it binds the terminal repeats (TRs) of the KSHV DNA to the genome of the host cell, specifically in histones H2A and H2B, thereby ensuring viral replication and effective segregation of the daughter cells [136,137]. For its part, LANA expression promotes chromosomal instability by inhibiting the activity of p53 and facilitating entry into the S phase [138]. LANA also interacts with the retinoblastoma protein (Rb) and acts as a transcription cofactor in the Rb/E2F regulatory pathway [139]. LANA also promotes overexpression and induces stabilization of β-catenin by binding to glycogen synthase kinase-3β (GSK-3β) and causing nuclear accumulation [140]. It increases the activity of human telomerase reverse transcriptase (hTERT) and promotes cell immortalization [141]. It downregulates the TGF-β type II receptor, promoting cell growth and inhibiting apoptosis by epigenetic mechanisms [142]. Finally, it contributes to angiogenesis by acting as a component of the ubiquitin complex and by targeting the von Hippel–Lindau protein (VHL) for degradation via the proteasome [143].

The balance between the KSHV latent and lytic viral life cycles is critical and is under the strict control of mechanisms that make it possible for a herpesvirus to get in and out of its host freely [144]. The lytic phase is necessary for viral spreading and it may take place immediately after the primary infection or after the lytic reactivation of a latently infected cell [135], inducing the expression of immediate-early, early, and late genes [145]. The lytic cycle plays an essential role in KS pathogenesis and development by stimulating paracrine angiogenesis through G protein-coupled receptors (GPCRs) via VEGF secretion [146].

There are several ways to induce change in the KSHV cycle. Studies with artificial inductors such as 12-*O*-tetradecanoylphorbol-13-acetate (TPA) have revealed the importance of epigenetic regulation and of the MAPK signaling pathway [147,148]. Reactivation can also be induced by multiple cellular factors, including intracellular immobilization of calcium, inhibition of the NF-κB pathway by lytic proteins, coinfection with other viruses, hypoxia, and oxidative stress [144].

An immediate-early gene encoded by ORF50 and homolog of RTA (a transcriptional activator encoded by the Epstein–Barr virus) activates the lytic cycle gene expression from the latent viral genome [149]. The DNA-binding domain allows RTA to directly bind and activate numerous viral promoters and the two KSHV origins of lytic replication (OriLyt-L and OriLyt-R), and the activation domain allows RTA to interact with cellular transcription factors and chromatin modification complexes to promote viral gene transcription [135]. RTA is necessary and sufficient to drive KSHV lytic replication and the production of viral progeny [150]. It activates different viral promoters through multiple mechanisms, including direct binding to DNA by recognizing RTA-responsive elements (RREs) [151]. Furthermore, RTA can transactivate essential early genes via interactions with cofactors such as RBP-Jk and Oct-1 and the viral Mta protein [152].

Upon completion of RTA-dependent viral replication, late gene synthesis proceeds by expressing structural proteins and glycoproteins, such as K8.1 glycoprotein. Finally, infectious virions are assembled that subsequently disseminate to other individuals [153]. LANA, activated in turn by RTA, interacts physically with its promoter, modulating its activity and reducing the production of KSHV virions [154]. Therefore, the balance between LANA and RTA largely controls the shift between latent and lytic infection.

### 4.3. Kaposi’s Sarcoma

#### Epidemiology of HHV8 Infection

**Figure 3:**
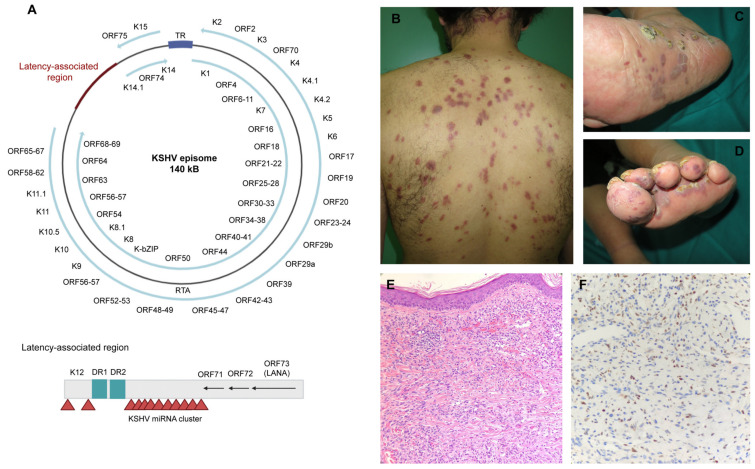
Human herpesvirus 8 and Kaposi’s sarcoma: (**A**) Scheme of HHV8. (**B**–**D**) The clinical aspect of Kaposi’s sarcoma, consistent with erythematous nodules located in the trunk of an HIV+ male (**B**) and in the foot of an elderly woman (**C**,**D**). (**E**) The H&E aspect. (**F**) The positive stain for HHV8. All the photographs were generated and provided by the authors.

HHV8 is responsible for Kaposi’s sarcoma (Figure 3). Kaposi’s sarcoma (KS) is a low-grade vascular tumor associated with Kaposi’s sarcoma herpesvirus/human herpesvirus 8 (KSHV/HHV8) infection. HHV8 shows significant differences from the other herpesvirus types whose seroprevalence is almost universal among adults. Three patterns have been identified: (1) high endemic areas, with >25% seroprevalence (including many regions in Africa); (2) mid-endemic areas, with 10–25% seroprevalence (e.g., the Mediterranean Basin); (3) non-endemic areas, with <10% seroprevalence. There are also regional and intranational variants [155]. Although the seroprevalence is <5% in the general population, it is much higher among men who have sex with men (MSM) (25–60% in HIV+ MSM and 20–30% in HIV– MSM) [156].

Understanding the transmission mechanisms of the virus was a challenge in the years immediately after its identification. Considering the epidemiological context of the epidemic outbreaks of Kaposi’s sarcoma with regard to HIV, sexual transmission was considered as a hypothesis [157,158,159], and it was suggested that, in particular, the virus spreads through homosexual relations between men. Subsequent studies showed that the virus was released through the mucosa, with frequent and asymptomatic viral release in immunocompetent men [160,161,162,163]. Therefore, the hypotheses about the transmission mechanisms of HHV8 moved beyond considering that it was solely transmitted through sexual relations between men. Since it was already known that HIV was not transmitted through saliva, this transmission mechanism represented an uncontrolled means for the virus to spread during the first years of the HIV pandemic. Saliva exchange seems to be a relevant factor in the transmission of HHV8 (KSHV). KSHV sequences may be detected in saliva, which seems to be the main transmission vehicle in MSM in non-endemic areas and in children in endemic areas [164]. In fact, saliva exchange during certain sexual activities seems to be the most important transmission mechanism among MSM; levels of the virus in semen, when detected, are much lower than those in saliva [164]. The transmission of the virus through blood or derivatives is possible since people infected with HHV8 may present the virus in their circulating mononuclear cells [165]. However, this type of transmission is generally rare, as is shown by the KS rate that is significantly lower among American patients who contracted HIV through the use of parenteral drugs. 

The risk factors associated with the development of KS and HHV8-related malignant lesions are age (whereby risk increases with age) and an immunocompromised status (caused by HIV, drugs, or the host’s genetic factors) [121].

Clinical features: Kaposi’s sarcoma lesions are predominantly present in the skin and mucosa, mostly affecting lower extremities, face, trunk, genitalia, and oropharyngeal mucosa, but in severe cases may also involve lymph nodes and visceral organs, including the respiratory and gastrointestinal tracts [166]. From a clinical perspective, the lesions are violaceous asymptomatic plaques and papules, which are customarily located on the limbs of elderly patients. In immunocompromised patients, including those with AIDS, KS may be more severe and affect the mucosa, including the palate, the pharynx and the larynx, and sometimes the gastrointestinal tract, in which case patients may present fecal blood. The more severe forms of KS are rare, affect the lungs, and have a poor prognosis. Four different epidemiologic/clinical variants of KS have been described [167]: (1) Classic type, more prevalent among Mediterranean elderly men. Its course is generally indolent. (2) Endemic African form, occurring primarily in young black men. It follows a more aggressive course than classic KS, especially the lymphadenopathic form. (3) Iatrogenic form associated with transplantation or drug immunosuppression. It occurs mainly in renal transplant recipients and, infrequently, after other solid organ or bone marrow transplantation. Transplant-associated KS has a protracted but aggressive course. In transplant recipients, KS lesions may regress after discontinuation of immunosuppressive therapy. (4) Epidemic HIV-associated form.

KS is an AIDS-defining illness and remains the most prevalent malignancy among patients with AIDS. In HIV-infected persons, AIDS–KS is a much more aggressive disease that typically manifests itself with disseminated lesions and visceral involvement. Kaposi’s sarcoma exhibits a less aggressive presentation in patients already receiving highly active antiretroviral therapy (HAART). Exacerbation (also called KS flare) can occur after therapy (e.g., corticosteroids, rituximab) or subsequently to the immune reconstitution inflammatory syndrome that may occur when initiating HAART in HIV-infected persons [168]. 

All these forms have common virological, histological, and clinical features [167]. Kaposi’s sarcoma manifests as violaceous asymptomatic lesions which evolve from early macules (patch stage) into plaques (plaque stage) that may grow into larger nodules (tumor stage). Different stages can coexist in the same individual at the same time. Newer histologic variants include anaplastic, hyperkeratotic, lymphangioma-like, bullous, telangiectatic, ecchymotic, keloidal, pyogenic granuloma-like, micronodular, intravascular, glomeruloid, and pigmented KS, as well as KS with sarcoid-like granulomas and KS with myoid nodules. Latency-associated nuclear antigen (HHV8) is the most specific immunohistochemical marker available to help distinguish KS from similar pathologies [169]. 

Treatment: The disease can be managed expectantly, or the treatment may be physical (e.g., cryotherapy or CO_2_ laser) or topical (e.g., imiquimod or photodynamic therapy). In the more severe forms, anthracycline-based regimens (generally liposomal doxorubicin) are the treatment of choice. The expectant approach is taken for mild classic cases. In immunocompromised or HIV+ patients, improving the immunocompromising conditions by treating HIV is the best strategy [170].

## 5. Conclusions

Several viral agents have been associated with different forms of skin cancer. There is clear evidence on the pathogenetic implication of certain types of HPV in cervical cancer, but also in anal, vulvar, penile carcinomas and in verrucous carcinoma of the anogenital region. Moreover, HPV5 and HPV8 have been associated with cSCC in individuals with epidermodysplasia verruciformis, and HPV has been associated with cSCC in immunocompromised patients, and some evidence has postulated its potential association with cSCC in immunocompetent individuals. Merkel cell polyomavirus has been linked to the development of Merkel cell carcinoma more recently, and HHV8 is responsible for Kaposi’s sarcoma. Proper knowledge of the mechanisms underlying these forms of cancer open opportunities for prevention in the future.

## Figures and Tables

**Table 1:** Major known HPV genera.

**Alpha**	Skin and mucosa	High-risk types	Pre- and malignant lesions (immortalize human keratinocytes)	HPV16	Cervical squamous cell carcinoma (~50) Cervical adenocarcinoma (~35) Oropharyngeal cancer (~25)
				HPV18	Cervical squamous cell carcinoma (~20) Cervical adenocarcinoma (~35)
				HPV31, 33, 35, 39, 45, 51, 52, 56, 58, 59	Cervical squamous cell carcinoma (~30)
		Low-risk types	Benign lesions (do not immortalize human keratinocytes)	HPV6, 11	Benign genital lesions Respiratory papillomatosis
				HPV13, 32	Oral focal epithelial hyperplasia
				HPV2, 3, 27, 57	Skin warts
**Beta**	Skin	Latent infections in the general population activated under conditions of immune suppression. Strangely, these viruses can cause epidermodysplasia verruciformis (EV), an aggressive growth of benign and malignant neoplasias of the skin, in genetically predisposed individuals		HPV5, 8	First beta HPV types isolated from SCC of EV individuals
				HPV9, 12, 14, 15, 17, 19–25, 36–38, 47, 49, 75, 76, 80, 92, 93, 96, 98–100, 104, 105, 107, 110, 111, 113, 115, 118, 120, 122, 124, 143, 145, 150–152, 159	Likely associated with SCC in EV patients as well as in immunocompromised and immunocompetent individuals
**Gamma**	Skin	Benign lesions. Histologically distinguishable by intracytoplasmic inclusion bodies specific to type species		HPV4, 48, 50, 60, 65, 88, 95, 101, 103, 108, 109, 112, 115, 116, 119, 121, 123, 126–142, 144, 146–149, 153–158, 161–170	Skin warts and papilomas
**Mu**	Skin and mucosa	Mostly cause clinically latent infections. Histologically distinguishable by intracytoplasmic inclusion bodies specific to type species		HPV1, 63, 204	Palmoplantar warts
**Nu**	Skin	Benign and malignant lesions		HPV41	The first and the only member of a new subgroup of HPVs. It has been detected in skin warts, but also in skin carcinomas and premalignant keratoses
